# Valorization of Agroindustrial Waste: Use of Adsorbents from Peanut Shells for Removal of Paracetamol and Propranolol

**DOI:** 10.1021/acsomega.6c03834

**Published:** 2026-09-15

**Authors:** Raissa A. Freitas, Ingrid L. S. Santana, Marina N. S. Silva, Giovanna A. J. C. Guedes, Carolaine O. L. Silva, João C. C. Rangel, Deivson C. S. Sales, Marta M. M. B. Duarte

**Affiliations:** † Departamento de Engenharia Química, 28116 Universidade Federal de Pernambuco, Recife 50670-901, Pernambuco, Brazil; ‡ Laboratório de Biocombustíveis (LAC/i-LITPEG), Universidade Federal de Pernambuco, Recife 50740-550, Pernambuco, Brazil; § Escola Politécnica de Pernambuco, 117110 Universidade de Pernambuco, Recife 50720-001, Pernambuco, Brazil

## Abstract

The improper disposal of pharmaceuticals such as paracetamol and propranolol represent a significant environmental concern due to their high persistence in the environment and low removal efficiency by conventional wastewater treatment processes. In this context, this work aimed to develop and evaluate five adsorbents derived from peanut shells precursor for removal of paracetamol and propranolol in a binary mixture (228 and 279 nm), an application that has not yet been explored for this bioresidue. Among the produced adsorbents, phosphoric-acid-modified activated carbon (CA_H_3_PO_4__) presented the best performance, achieving removal efficiencies of 99.40% and 99.47% at wavelengths of 228 and 279 nm, respectively. This result can be attributed to its high specific surface area (547 m^2^ g^*–*1^) and the presence of oxygenated and phosphorylated functional groups on the CA_H_3_PO_4__ surface, formed through thermochemical treatment. The kinetic data were best fitted to pseudo-second order model, indicating the predominance of chemical interactions between adsorbate and adsorbent. In the equilibrium study, the data best fitted the Sips model, suggesting a surface with both homogeneous and heterogeneous adsorption sites. Thermodynamic analysis revealed that the adsorption process is exothermic, indicating spontaneous and energetically favorable interactions. These results highlight the high adsorption potential of CA_H_3_PO_4__ as an effective and viable alternative for the treatment of pharmaceutical-contaminated effluents.

## Graphical abstract

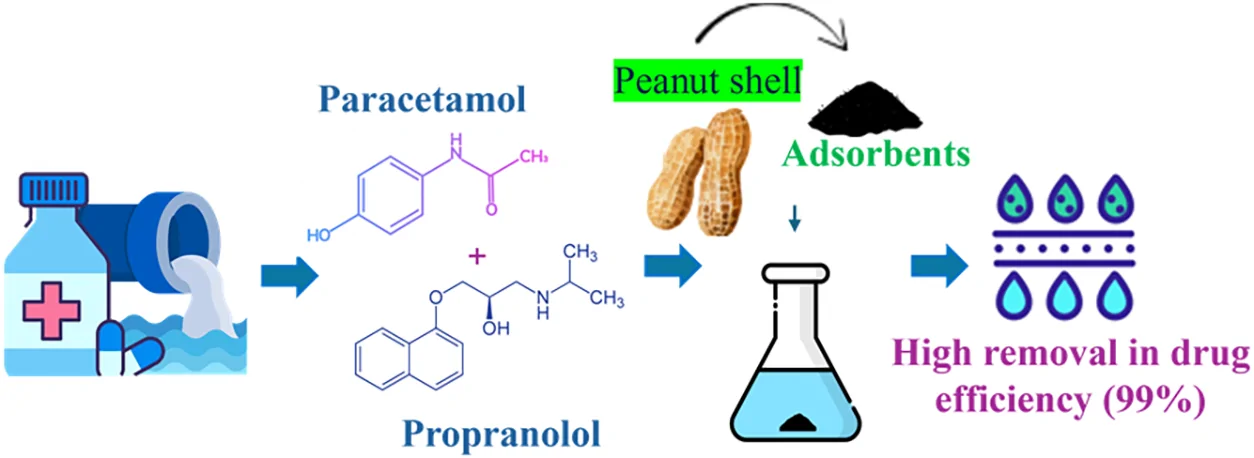

## Introduction

The presence of pharmaceuticals in aquatic environments is a significant environmental concern due to their potential damage to ecosystems and human health.^1,2^ Studies have also demonstrated the bioaccumulation of these substances in organs and muscle tissues of different species of fish and other aquatic organisms.^3^

Among these compounds, paracetamol stands out as widely recommended because of its low cost and wide access and availability.^4^ Another frequently detected substance is propranolol, which is one of the most prescribed β-blockers for the treatment of arterial hypertension.^5^ These pharmaceuticals and their metabolites reach the environment mainly through the improper disposal of domestic sewage, industrial effluents, and hospital waste. This practice promotes the pollution of water bodies, since these substances are recalcitrant, and conventional treatment methods have limited efficiency in their removal.^6^

Because of these limitations, alternative wastewater treatment methods have been investigated to promote an efficient removal of this type of contaminant. Such processes include advanced oxidation,^7,8^ adsorption,^9–12^ and photocatalytic degradation.^13,14^ In this way, adsorption stands out for its high efficiency, operational versatility, potential for using waste materials as adsorbent sources, and easy integration with conventional treatment systems.^15^

Adsorbent materials derived from biomass and agroindustrial waste have been extensively studied for the removal of inorganic and organic compounds^16–18^ since they present high carbon levels and low ash content, characteristics that favor their removal capacity, presenting high removal and regeneration capacity according to Selmi et al.^19^

Several precursors have been used for the preparation of adsorbents, aiming at the removal of pharmaceuticals from aqueous media, such as bamboo residues,^20^ pistachio shells,^21^ moringa oleifera seeds,^22^ and coffee husks.^23^ Another promising bioresidue that can be used as a precursor for the development of adsorbents is peanut shells, due to its high availability and underutilization. The United States Department of Agriculture (USDA) reports that global peanut production in the 2024/2025 crop year totaled 50.53 million tons, generating approximately 15.46 million tons of shells.^24^ This residue is generally used as fuel (burned), in soil fertilization, or as animal feed; however, most of it is discarded without proper management.

Although the use of peanut shells as a precursor for preparing adsorbents to remove pharmaceuticals from aqueous media has been widely reported in the literature,^25–27^ most of these studies are limited to evaluating systems containing only a single pharmaceutical. However, effluents typically present complex matrices containing two or more contaminants, which can lead to antagonistic, synergistic, or noninteractive effects-factors that directly impact the adsorption capacity for the contaminants under evaluation.^28^ Therefore, investigating the adsorbent’s performance for adsorption of pharmaceuticals in multicomponent systems contributes to advancing knowledge about adsorption behavior and to developing more effective strategies for treating water contaminated by pharmaceuticals.

Thus, this study introduces an adsorbent-adsorbate combination not yet explored in the literature as an innovation, aiming to contribute to the evaluation of the adsorption process for removing the pharmaceuticals paracetamol and propranolol in a multicomponent system using peanut shells as a precursor for adsorbent preparation. The specific objectives of this study were (I) to prepare five different adsorbents using peanut shells as the precursor; (II) to select, in a preliminary study, the adsorbent to be used based on adsorption capacity and removal percentage values; (III) to characterize the selected adsorbent and its precursor using Fourier transform infrared spectrophotometry (FT-IR), pH at the point of zero charge (pH_PZC_), scanning electron microscopy, X-ray diffraction, and determination of surface area, pore diameter, and pore volume; (IV) to determine adsorption conditions by evaluating the effects of initial pH, adsorbent dosage, and stirring speed; (V) to evaluate adsorption kinetics and equilibrium by applying mathematical models; (VI) to study thermodynamic properties; and (VII) to conduct a comparative study with commercial activated carbon.

## Methodology

### Preparation of Solutions

Stock solutions of 1000 mg L^*–*1^ were prepared using paracetamol (acronym: PCM; molecular formula: C_8_H_9_NO_2_; purity: 99%) and propranolol (acronym: PPN; molecular formula: C_16_H_21_NO_2_; purity: 99%). In sequence, binary mixtures (BM) were prepared from each stock solution at 10 mg L^*–*1^ in distilled water. The concentrations of BM were determined before and after the experiments using UV/Vis spectrophotometry (brand: Genesys; model: Genesys 10S; wavelength: 228 and 279 nm, selected based on the maximum absorption peaks observed in the BM spectrum, as shown in Figure S1).

During the analysis, the characteristic wavelengths of the pharmaceutical mixture were monitored, and analytical calibration curves (Figure S1) were built for each identified wavelength. These curves were evaluated in terms of linearity and precision based on the correlation coefficient (r), the coefficient of variation (CV), and residual error. From this evaluation, the limits of detection (LOD) and the limit of quantification (LOQ) were determined (Table S1). The results indicated that the curves meet the linearity and precision criteria established by the Instituto Nacional de Metrologia, Qualidade, e Tecnologia (INMETRO).^29^ INMETRO is the Brazilian institution responsible for establishing guidelines regarding metrological quality and the validation of analytical methods, ensuring the reliability and traceability of the results obtained.

### Preparation of Adsorbents

In the preparation of adsorbents were used sodium hydroxide (molecular formula: NaOH; brand: Química Moderna; purity: 99%), nitric acid (molecular formula: HNO_3_; brand: Química Moderna; purity: 99%), phosphoric acid (molecular formula: H_3_PO_4_; brand: Química Moderna; purity: 97%), sodium bicarbonate (molecular formula: NaHCO_3_; brand: Vetec; purity: 95%), and hydrogen peroxide (molecular formula: H_2_O_2_; brand: Neon; volume: 35%).

Peanut shells were washed with running water and oven-dried (brand: Quimis; model: Q317M22) at 378 K for 24 h. After drying, the material was ground in a knife mill (brand: Cielab; model: CE-430), washed with distilled water, and oven-dried again, yielding the *in natura* (IN) adsorbent. The IN adsorbent was fractionated and subjected to four types of treatments: alkaline washing with sodium hydroxide (BW), chemical activation with phosphoric acid (CA_H_3_PO_4__), physical activation with carbon dioxide (CA_CO_2__), and functionalization with hydrogen peroxide (FC), whose preparation steps are shown in Figure 1. The step-by-step preparation procedures for each adsorbent also can be found in Supporting Information S2. The adsorbents produced were sieved and classified with particle sizes smaller than 0.090 mm, according to Silva et al.^30^ A particle size of less than 0.090 mm, although potentially requiring specific strategies for adsorbent separation and recovery in large-scale systems, enhances the adsorption performance and represents a promising characteristic for the development and optimization of wastewater treatment applications.

**Figure 1:**
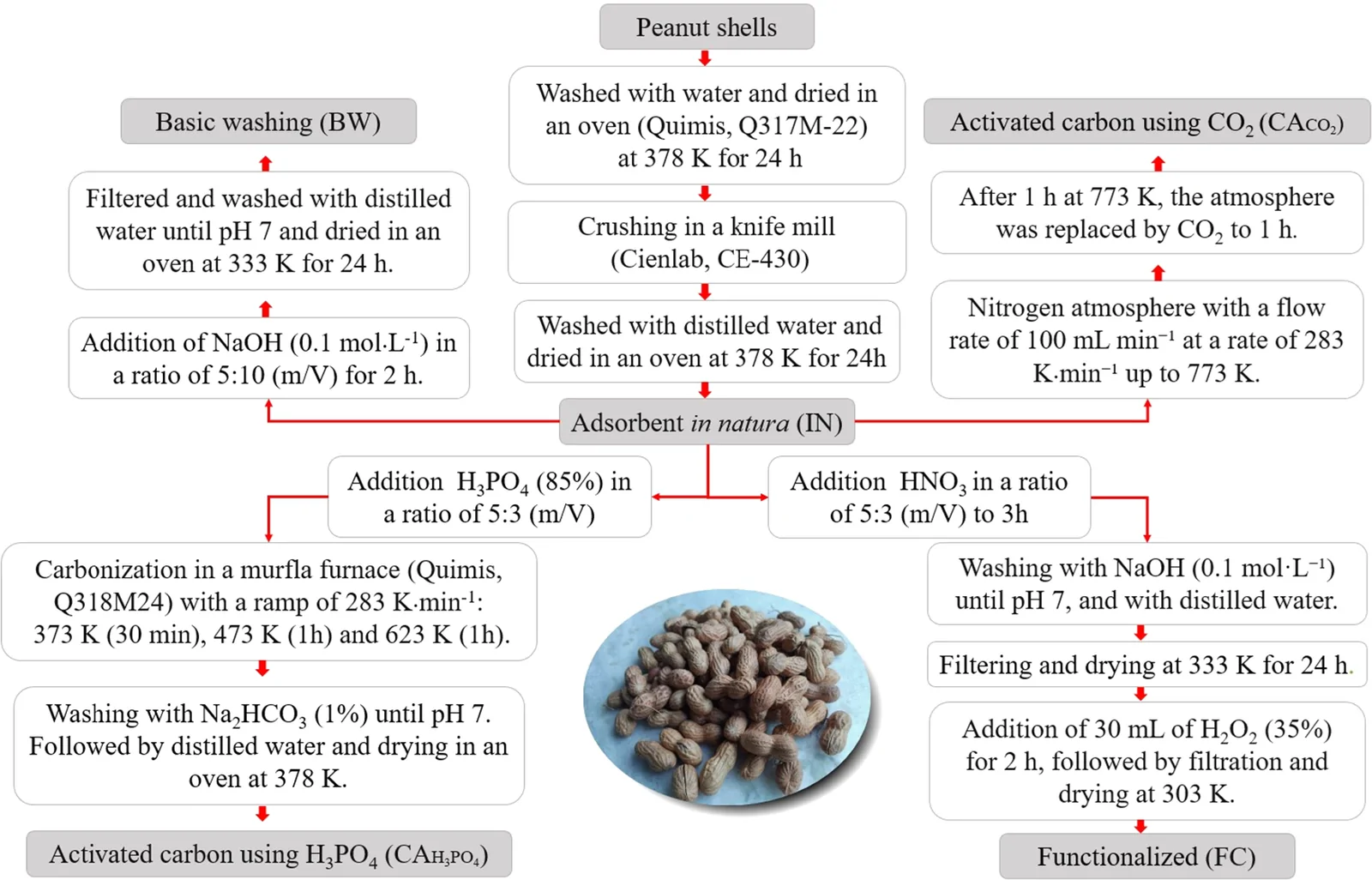
Preparation steps of the adsorbents from peanut shells.

Following the preparation of the adsorbents as shown in Figure 1, a comparative evaluation was conducted to select the adsorbent that demonstrated the best performance in the adsorption of the drug mixture.

### Preliminary Evaluation

Preliminary experiments were performed by adding 0.1 g of each adsorbent separately to 50 mL of a binary mixture solution. The suspensions were placed in a shaker incubator (brand: Splabor; model: SP-223) under 100 rpm at 298 ± 2 K and pH 5.5 (natural of solution) for 120 min. In sequence, the suspensions were filtered using a quantitative filter paper (brand: Química Moderna; type: blue ribbon) and quantified. Blank experiments were also performed. The experiments were performed in quintuplicate.

The efficiency of adsorbents was evaluated by adsorption capacity (*q*) (mg g^*–*1^) and percentage of removal (*R*) (%) according to eqs 1 and 2, respectively.

1 $$q=\frac{\left(C_{0}-C\right)}{V}$$

2 $$R=100\left(\frac{C_{0}-C}{C_{0}}\right)$$

where *C*_0_ and *C* (mg L^*–*1^) are the initial and final solute concentrations in the solution, respectively; *V* (m^3^) is the volume of solution, and *m* (g) is the mass of adsorbent.

### Characterization of Selected Adsorbent

The selected adsorbent and its precursor were characterized by Fourier Transform Infrared Spectrophotometry (FT-IR) analysis (brand: Bruker; model: Tensor 27; method: Attenuated Total Reflection (ATR); wavenumber: 400–4000 cm^*–*1^; resolution: 4 cm^*–*1^) to identify of the functional groups present in the structures of materials.

The experiments for pH of the point of zero charge (pH_PZC_) determination were performed using 0.1 g of adsorbent, which was kept in contact with 25 mL of distilled water for a pH ranging from 2 to 10. The suspensions were stirred in a shake incubator (brand: Splabor; model: SP-223) under 100 rpm at 298 ± 2 K for 24 h. The pH of the solutions was adjusted with 0.1 mol L^*–*1^ sodium hydroxide (molecular formula: NaOH) and 0.1 mol L^*–*1^ hydrochloric acid (molecular formula: HCl) solutions, measured using a pHmeter (brand: Quimis; model: Q488AS). The measurement was repeated after 24 h.

The surface morphologies of the IN and CA_H_3_PO_4__ adsorbents were evaluated using a scanning electron microscope (brand: TESCAN; model: VEGA3; accelerating voltage: 5 kV; magnification: 20,000×) equipped with a thermionic emission source and a tungsten filament. The samples were metal-coated and mounted on a holder by using double-sided tape for analysis.

X-ray diffractograms were obtained using an X-ray diffractometer (brand: BRUKER; model: D2-PHASER; source: Cu–Kα; wavelength: 0.15406 nm; 2θ: 20–80°; Δθ: 0.5°; scan speed: 1.2° min^*–*1^). A 1 mm convergent beam, a 3 mm air-scatter slit, and a 0.5 mm Ni filter were used to minimize Fe fluorescence effects.

The specific surface area (*S*_BET_) and pore volume (*V*_pore_) were determined by the Brunauer–Emmett–Teller (BET) method. Additionally, the average pore diameter (*d*_pore_) and pore size distribution were obtained by the Barrett–Joyner–Halenda (BJH) method. For the analysis, a surface area and pore size analyzer (brand: Quantachrome; model: Nova-2000; gas: N_2_; relative pressure: 0–0.3) was used. The IN material was heated to 323 K, while the CA_H_3_PO_4__ was heated to 393 K. Both materials were heated under a vacuum for 6 h.

### Evaluation of Adsorption Conditions

For the selected adsorbent, adsorption experiments were performed in a finite bath system using the shaker incubator for 120 min. The effect of initial pH of binary mixtures was evaluated for pH 3.5, 4.5, and 5.5 (values based on pH_PZC_ analysis) for a constant adsorbent dosage (*m*/*V*) of 2.0 g L^*–*1^ under 100 rpm. The experiments were performed in triplicate. Subsequently, at the optimum pH value determined by previous study, the effect of adsorbent dosage was evaluated for 1.0, 2.0, 4.0, 8.0, and 16.0 g L^*–*1^ under 100 rpm. The experiments were performed in duplicate. Finally, the effect of different stirring speeds of 50, 100, 150, 200, and 250 rpm were also evaluated in the optimum pH and adsorbent dosage values determined previously. For all experiments, the temperature was maintained as 298 ± 2 K. The experiments were performed in triplicate.

### Evaluation of Adsorption Kinetics

The kinetic evolution of the adsorption process was evaluated under the conditions established in the previous study for a time interval from 0 to 120 min. The experiments were performed in triplicate. The experimental data were fitted to pseudo-first-order (PFO),^31^ pseudo-second-order (PSO),^32^ Weber–Morris,^33^ and Boyd^34^ models (Table S2) using the software Origin 7.5 (OriginLab Corporation, MA). The determination coefficient (*R* ^2^) and residual sum of squares (RSS) were determined.

### Adsorption Equilibrium and Thermodynamics Evaluation

In the adsorption equilibrium evaluation, different initial concentrations of mixtures were investigated ranging from 1 to 600 mg L^*–*1^ for the equilibrium time defined by the kinetic study at 298 ± 2 K. The experiments were performed in duplicate. Subsequently, the experimental data were fitted to the Langmuir,^35^ Freundlich,^36^ and Sips^37^ models (Table S3). The determination coefficient (*R* ^2^) and the residual sum of squares (RSS) also were determined.

The thermodynamic study was performed under the same conditions as the adsorption equilibrium study at temperatures of 298 ± 2, 313 ± 2, and 328 ± 2 K. Subsequently, the variations of Gibbs free energy (Δ*G*), and enthalpy (Δ*H*), entropy (Δ*S*) were determined according to Abumelha et al. (Table S4).^38^ The linearized thermodynamic plots are available in Figure S2.

### Comparative Evaluation

The adsorption capacity and percentage of removal of the selected adsorbent were compared with those presented by a commercial adsorbent previously characterized by Silva et al.^39^ for which the adsorption of the pharmaceuticals was performed under the same conditions as the kinetic study carried out with the produced adsorbent. The experiments were performed in quintuplicate.

## Results and Discussion

### Preliminary Evaluation

The FC material presented no contaminant removal capacity at any of the evaluated wavelengths. Conversely, the IN and BW adsorbents demonstrated substantial contaminants removal at 228 nm. Table 1 shows the adsorption capacity (*q*) and percentage of removal (*R*) values obtained from preliminary experiments with the adsorbents.

**Table 1:** Preliminary Results Obtained by Using Adsorbents Prepared from Peanut Shell

	228 nm		279 nm	
adsorbent	*q* (mg g^–1^)	*R* (%)	*q* (mg g^–1^)	*R* (%)
IN	1.09 ± 0.02	21.12 ± 0.78	NA^a^	0
BW	2.01 ± 0.04	38.77 ± 0.77	NA	0
CA_H_3_PO_4__	4.61 ± 0.06	94.88 ± 1.13	4.30 ± 0.02	87.39 ± 0.43
CA_CO_2__	1.56 ± 0.02	3.142 ± 0.32	0.74 ± 0.03	15.26 ± 0.63
FC	NA	0	NA	0

a NA = Not adsorbed.

According to the results presented in Table 1, CA_CO_2__ and CA_H_3_PO_4__ removed the pharmaceuticals at both evaluated wavelengths; however CA_H_3_PO_4__ presented the highest removal efficiency. Similar result was reported by de Lima et al.,^40^ who prepared activated carbons using H_3_PO_4_ (550 and 650 °C) from invasive plants *(Rhododendron ponticum*). The authors observed that the activation process led to an increase of approximately 50% in the removal efficiency.

The increase in the adsorption capacity of materials subjected to acid activation is associated with the greater generation of oxygenated functional groups on the adsorbent surface. Additionally, the chemical agents employed act as dehydrating agents, promoting the development of porosity, which results in an increase in the surface area. In this context, CA_H_3_PO_4__ was selected for further study.

### Characterization of Selected Adsorbent

CA_H_3_PO_4__ and its precursor (IN) were characterized according to item 2.4. The FT-IR spectra and pH_PZC_, SEM and EDS for CA_H_3_PO_4__ and IN materials are shown in Figure 2.

**Figure 2:**
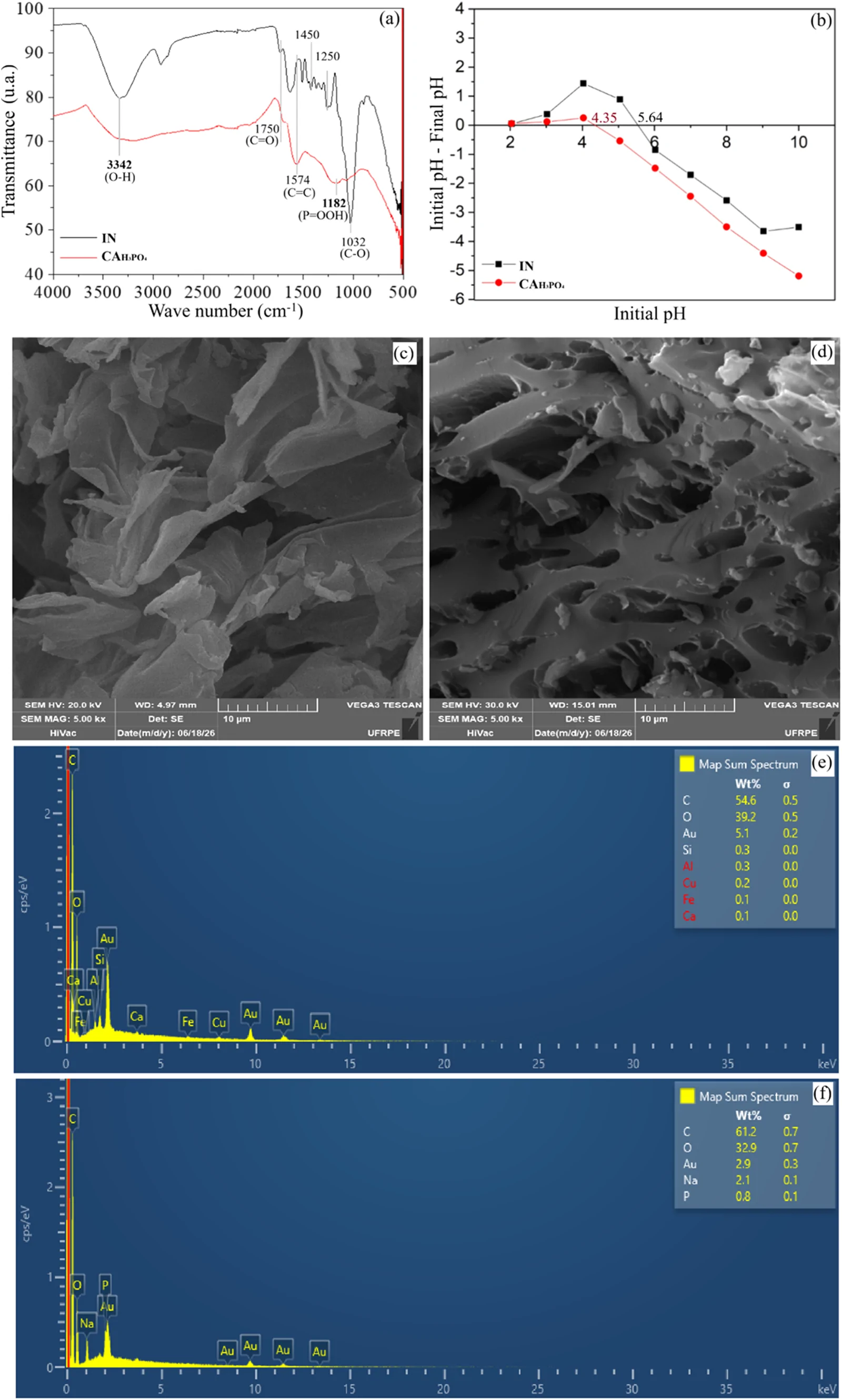
Characterization of selected adsorbent: (a) FT-IR spectra, (b) pH_PZC_ for CA_H_3_PO_4__ and IN adsorbents, (c) SEM for IN, (d) SEM for CA_H_3_PO_4__, (e) EDS for IN, and (f) EDS for CA_H_3_PO_4__.

The FT-IR spectra presented in Figure 2a indicate reduction in the number and/or a shift of the characteristic peaks of functional groups present on the surface of the IN compared to CA_H_3_PO_4__ due to structural modifications associated with thermochemical treatment.

Functional groups related to the lignocellulosic components present in the peanut shell were released due to activation process with peaks observed between 1574 and 1182 cm^*–*1^. The groups corresponding to the hemicellulose fraction (−COOH) and cellulose (−C–O) can be identified in the range of 1450–1250 cm^*–*1^, as well as the lignin fraction (−C–O) identified between 1750 and 1600 cm^*–*1^.^41^ The formation of these groups was also observed by Kerkhoff et al.^42^ in their studies about the production of biochar from *Butia capitata* endocarp. In addition, the peak at 1182 cm^*–*1^ can be attributed to the stretching vibrations of P=OOH or P–O–C bonds corresponding to phosphate groups, while the peak at 3342 cm^*–*1^ is attributed to the O–H stretching vibration of hydroxyl groups.^43^

As shown in Figure 2b, the pH_PZC_ value decreased from 5.64 for the IN to 4.35 after the activation process. This result confirms the acidic character of CA_H_3_PO_4__, since activation with H_3_PO_4_ promotes the formation of oxygenated groups, particularly carboxylic and phenolic groups, on the material surface, as previously observed in the FT-IR analysis. Considering that the pH of the working solution (pH = 5.5) is higher than the pH_PZC_ value of CA_H_3_PO_4__, the adsorbent surface is predominantly negatively charged, resulting in an anionic surface and thus favoring the adsorption of cationic species such as the pharmaceutical molecules studied.

The SEM micrograph, shown in Figure 2c,d, evidenced increase in the porosity of CA_H_3_PO_4__ compared to IN, indicating that the activation process promoted the removal of volatile components, thereby favoring the development and expansion of the material’s porous structure. EDS analysis, Figure 2e,f, revealed a higher proportion of carbon and oxygen in these materials. However, for the IN material, traces of Mg, Fe, Cu, and K were identified, which can be attributed to the intrinsic mineral composition of the plant biomass resulting from the absorption of these elements from the soil during plant growth. Furthermore, the presence of Si may be associated with the deposition of mineral particles from the soil or environment adhered to the biomass surface. These elements were not identified in CA_H_3_PO_4__, demonstrating the efficiency of the treatment process steps in modifying the surface composition of the material.

The X-ray diffraction (XRD) analysis (Figure S3) showed a broad and diffuse band in the 2θ region between 20° and 40° for both materials, which is characteristic of materials with a low degree of structural order and a predominance of amorphous phases. This behavior is typical for activated carbons obtained by H_3_PO_4_ activation, an agent that promotes the degradation of the crystalline regions of the precursor biomass and favors the formation of a porous carbonaceous matrix.

The pore size distribution diagram presented in Figure S4 exhibits an intense, narrow peak with pore sizes below 2 nm, indicating that the adsorbent structure is predominantly microporous, which corroborates the classification by Thommes et al.^44^ In addition, the presence of small adjacent peaks in the 2–4 nm range was also observed, which may be associated with a nonuniform pore distribution on the material’s surface.

In Table 2 is shown the specific surface area (*S*_BET_), pore volume (*V*_pore_), and average pore diameter (*d*_pore_) results obtained for IN and CA_H_3_PO_4__ adsorbents. Other results observed in the literature are included for comparison purposes.

**Table 2:** Specific Surface Area (*S*_BET_), Pore Volume (*V*_pore_), and Average Pore Diameter (*d*_pore_) Results Obtained for IN and CA_H_3_PO_4__ Adsorbents

adsorbent	*S*_BET_ (m^2^ g^–1^)	*V*_pore_ (cm^3^ g^–1^)	*d*_pore_ (nm)	ref
IN	2.10	0.005	9.5	this work
CA_H_3_PO_4__	547.00	0.020	2.2	this work
CAM^a^	254.91	-	2.3	^32^
PB-450^b^	911.59	0.070	-	^33^
CAS^c^	769.00	0.050	2.6	^34^

a CAM: activated carbon produced by chemical activation from castor bean cake at 1073 K.

b PB-450: activated carbon produced by chemical activation from banana peels at 723 K.

c CAS: activated carbon produced by chemical activation from soursop seeds at 623 K.

Based on Table 2, a reduction in the *d*_pore_ and an increase in both *S*_BET_ and *V*_pore_ values of the CA_H_3_PO_4__ were observed when compared to the IN. This structural change during the activation process is related to the decomposition of the lignocellulosic material, leading to the formation of cavities. During the carbonization step, the impregnated acid volatilizes, creating pores and externally interconnected cavities that ultimately form a network of meso- and micropores.^43^ Similar behavior has also been reported in previous studies,^45,46^ as well as in investigations using chemical activation.^39^

According to Santos et al.,^47^ who used the same materials for the adsorption of Cd^2+^ and Pb^2+^ ions, the N_2_ adsorption isotherms indicated type III behavior for IN and type I behavior for CA_H_3_PO_4__ adsorbents. Type III isotherms suggest a low adsorption affinity between the adsorbate and the material, whereas type I is associated with a high affinity, as evidenced by significant adsorption at low relative pressures.^44^ Furthermore, Santos et al.^47^ performed thermogravimetric analysis and Boehm titration on the materials. The authors observed that IN exhibited two stages of thermal decomposition: the first occurring below 473 K and the second between 473 and 723 K, with a DTG peak at 623 K, indicating that carbonization temperatures should not exceed this value. CA_H_3_PO_4__ also presented two decomposition stages, with the first mass loss occurring below 473 K and the second occurring between 673 and 1073 K. Boehm titration revealed that chemical activation led to an increase in basic, phenolic, and carboxylic functional groups, with carboxylic groups being predominant while lactonic groups remained unchanged.

### Evaluation of Adsorption Conditions

The results for the effect of the initial pH of the solution indicated a complete removal (approximately 100%) of the contaminants at evaluated pH values (Table S5). PCM is a weak electrolyte with a p*K*_a_ of 9.38 and can exist in ionized and nonionized forms. The distribution of these forms depends on the pH: up to pH 7, 90% of PCM is protonated; between pH 7 and 9, deprotonation occurs, making it neutral; and above pH 9.38, it assumes the anionic form.^48^ The same behavior was observed for PPN (p*K*_a_ of 9.45), which contains hydroxyl and secondary amino groups that can form anions and cations in solution.^49^ At the pH evaluated, both pharmaceuticals were protonated, thus they had a positive charge. The pH_PZC_ value for CA_H_3_PO_4__ was 4.35, indicating that its surface is negatively charged at pH 5.5, thereby favoring electrostatic attraction with the adsorbates. For subsequent evaluations, the natural pH of the solution (pH 5.5) was selected, which reduced operating costs.

Another important variable to consider is the adsorbent dosage as it directly affects both the adsorptive capacity and the removal percentage. The effect of adsorbent dosage (*m*/*V*) for adsorption of pharmaceuticals on CA_H_3_PO_4__ are shown in Figure 3.

**Figure 3:**
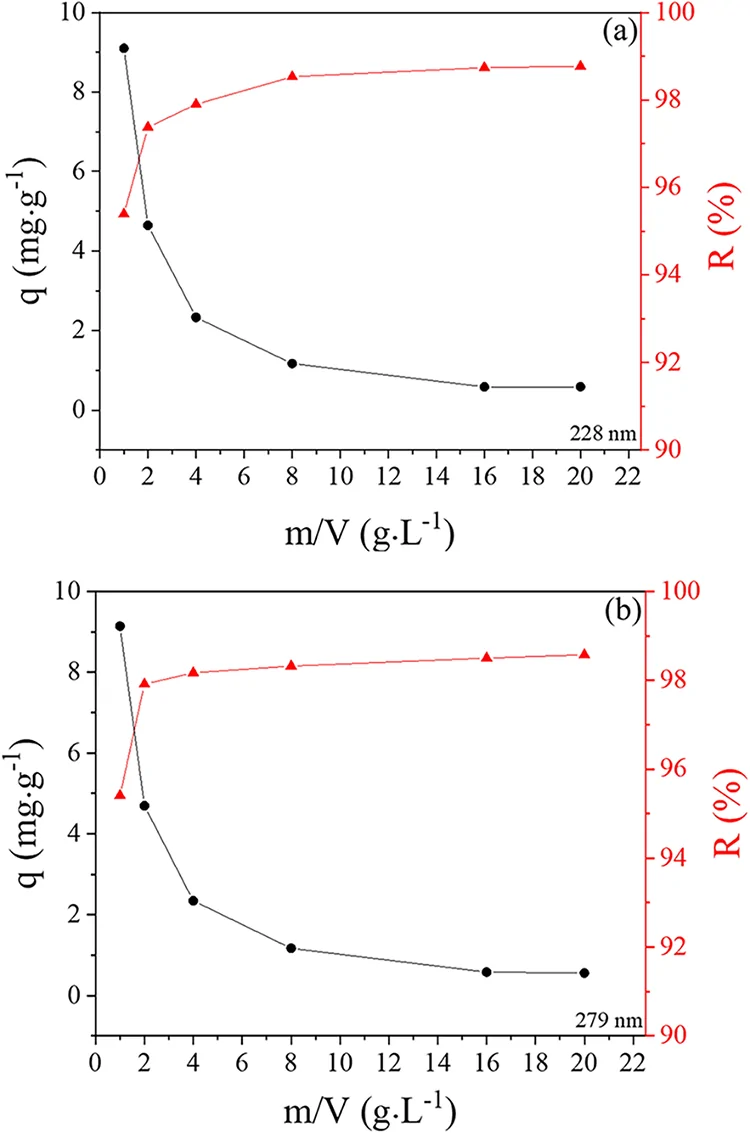
Effect of adsorbent dosage (*m*/*V*) for adsorption of pharmaceuticals on CA_H_3_PO_4__ at (a) 228 nm and (b) 279 nm.

From Figure 3, it was observed that the increase in the adsorbent dosage (*m*/*V*) implied an increase in the *R* until stabilization, as well as a decrease in *q*. Initially, an increase in the adsorbent dosage provides more active sites available for adsorption, which explains the increase in *R* value. On the other hand, this greater amount of free active sites promotes a reduction in *q* value.^50^ Since the *q* and the *R* are equally important for the process, the best operating condition of the process is the one in which both have the highest value simultaneously. Therefore, the adsorption dosage of 2 g L^–1^ was selected for the subsequent experiments.

The stirring speed (SS) (rpm) can influence the mass transfer of pharmaceutical molecules from the bulk solution to the adsorbent surface. The results of SS effect on pharmaceuticals adsorption are presented in Table 3.

**Table 3:** Effect of Stirring Speed on the Adsorption of a Binary Mixture of Pharmaceuticals on CA_H_3_PO_4__

	228 nm		279 nm	
SS (rpm)	*q* (mg g^–1^)	*R* (%)	*q* (mg g^–1^)	*R* (%)
50	4.67 ± 0.02	91.15 ± 0.31	4.41 ± 0.03	85.56 ± 0.64
100	4.52 ± 0.02	92.21 ± 0.43	3.97 ± 0.01	82.07 ± 0.57
150	4.64 ± 0.01	94.73 ± 0.09	4.30 ± 0.01	88.27 ± 0.09
200	4.61 ± 0.01	94.88 ± 0.11	4.13 ± 0.00	85.38 ± 0.05
250	5.03 ± 0.00	96.30 ± 0.04	4.58 ± 0.01	89.26 ± 0.17

According to Table 3, the difference between the lowest and highest *R* values was 5% for λ = 228 nm and 4% for λ = 279 nm. These results indicate that the adsorption process of the pharmaceuticals mixture studied onto CA_H_3_PO_4__ presented low external mass transfer limitation within the evaluated SS range. Therefore, adsorption is likely governed by other mechanisms, such as internal diffusion or adsorbent–adsorbate interactions. Based on these findings, an SS of 50 rpm was selected for subsequent experiments in order to reduce the energy consumption of the process. On the other hand, it is important to note that although 50 rpm has proven to be sufficient in low-viscosity aqueous solutions, in more complex real effluents, it may be necessary to adopt higher speeds to avoid mass transfer limitations.

### Evaluation of Adsorption Kinetics

The experimental adsorption data for the wavelength of 228 and 279 nm related to BM, together with the fitting of the pseudo-first order (PFO) and pseudo-second order (PSO) kinetic models, are presented in Figure 4a–c, respectively. To further complement the kinetic analysis, the intraparticle diffusion models of Weber–Morris^33^ and Boyd^34^ were applied, as shown in Figure 4d.

**Figure 4:**
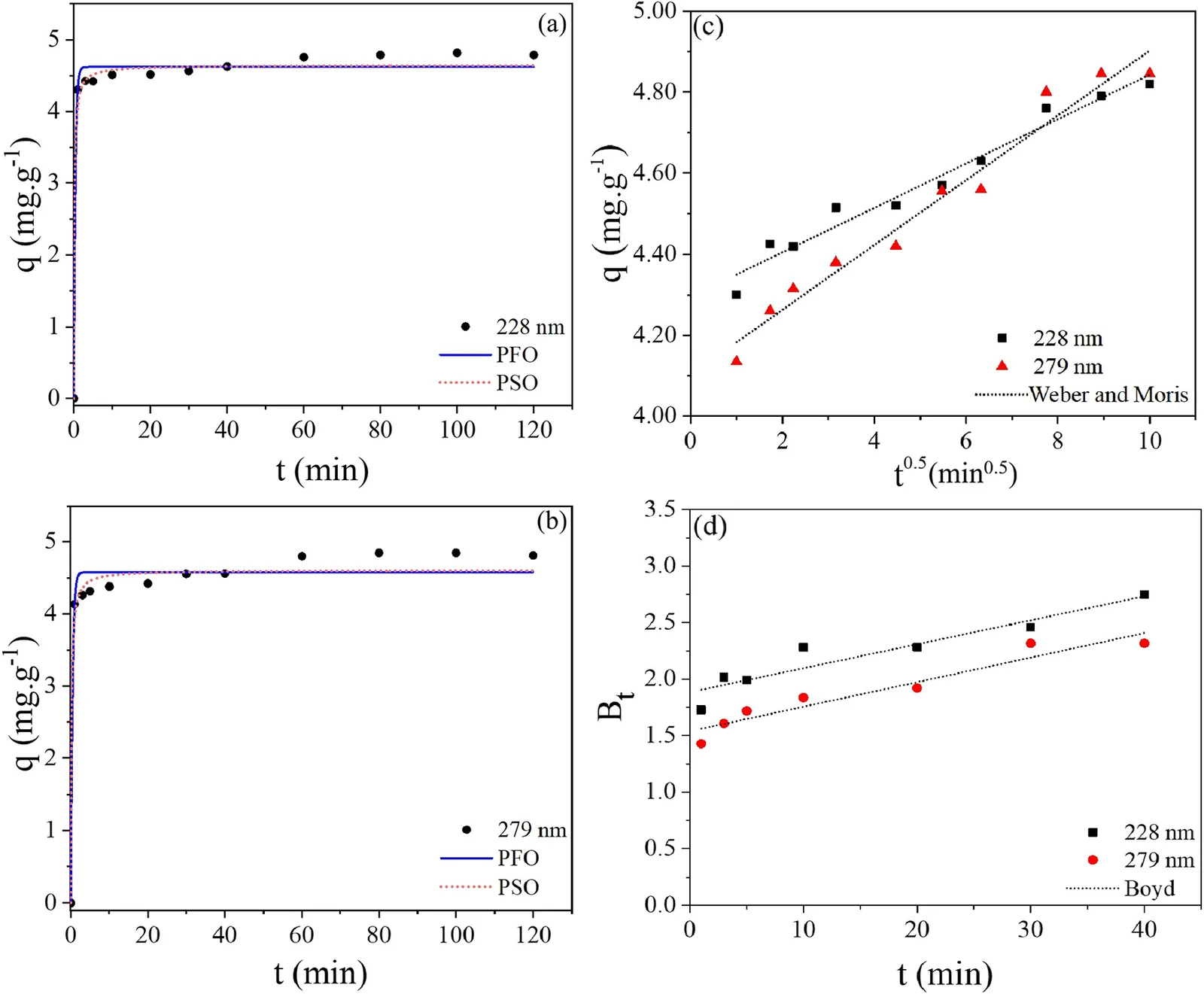
Fit of experimental data of contaminants adsorption on CA_H_3_PO_4__ to kinetic models at (a) 228 nm and (b) 279 nm and to intraparticle diffusion model of (c) Weber and Morris and (d) Boyd.

From Figure 4a,b, a rapid initial adsorption was observed for both wavelengths evaluated, with approximately 90% removal achieved within the first 3 min of contact. After this period, the adsorption rate decreased, and system stabilization occurred at around 60 min, reaching equilibrium with removal efficiencies of 98% and 99% for λ = 228 and 279 nm, respectively. The high initial removal can be attributed to the large availability of free active sites on the adsorbent surface, as well as to the presence of oxygen-containing functional groups formed during the treatment process. From Figure 4c, it was observed that the data are linear (*R* ^2^ ≥ 0.98) and do not intercept the origin, suggesting that the adsorption mechanism of pharmaceuticals on CA_H_3_PO_4__ is complex and that intraparticle diffusion is not the only rate-limiting step in the adsorption process (Table S6). This result suggests the action of other mechanisms involved, such as rapid adsorption on the surface and/or molecular interactions, which is in agreement with what was observed in the study of SS.^51^ It can be observed through Figure 4d that the fit of the experimental data to the Boyd model was similar to that obtained for the Weber and Morris model. The results indicate linear behavior with *R* ^2^ *>* 0.98.^34^ The estimated values of the unknown model parameters are presented in Table 4.

**Table 4:** Parameters of the PFO and PSO Kinetic Models for Adsorption of Mixture on CA_H_3_PO_4__

	PFO		PSO	
parameter	228 nm	279 nm	228 nm	279 nm
*q*_eq_ (mg g^–1^)	4.62 ± 0.05	4.58 ± 0.07	4.67 ± 0.04	4.65 ± 0.06
*k*_1_ (min^*–*1^)	2.65 ± 0.57	2.31 ± 0.50	-	-
*k*_2_ (g mg^–1^ min^*–*1^)	-	-	1.97 ± 0.60	1.30 ± 0.42
*R* ^2^	0.9889	0.9755	0.9931	0.9847
RSS	0.0218	0.0478	0.0136	0.0299

According to Table 4, *R* ^2^ values were similar; however, RSS was lower for PSO compared to the PFO model, suggesting that the adsorption of mixture by CA_H_3_PO_4__ follows PSO kinetics. Thus, the adsorption process is mainly governed by adsorbate-adsorbent interactions, suggesting that the rate-limiting step is associated with the occupation of active sites on the material’s surface, characterizing its driving force.^52^ Therefore, according to FT-IR analyses and the kinetic study, these interactions likely occur through C–P=OOH, C–P–O–C, or C–O groups and the cationic parts of the pharmaceuticals. Similar behavior was reported by Paixão et al.^53^ in the adsorption of PPN on phosphorus-doped mesoporous carbon. The authors report that the kinetics of pharmaceutical products are typically governed by the PSO model and controlled by these interactions.

Furthermore, the difference between calculated and experimental values of *q*_eq_ was 1.9% (λ = 228 nm) and 3.1% (λ = 279 nm) for PSO model, while for PFO it was 2.4% (λ = 228 nm) and 4.6% (λ = 279 nm). The PSO model also better described the adsorption of PCM in studies developed by Islam et al.^54^ in which palm leaf residues were used to produce activated carbon with KOH. This material presented a lower kinetic rate (*k*_2_ = 0.859 g mg^*–*1^ min^*–*1^) and a percentage of removal of 64% (*C*_0_ = 20 mg g^*–*1^) in 240 min.

### Adsorption Equilibrium and Thermodynamics Evaluation

The fit of adsorption models to experimental data are shown in Figure 5.

**Figure 5:**
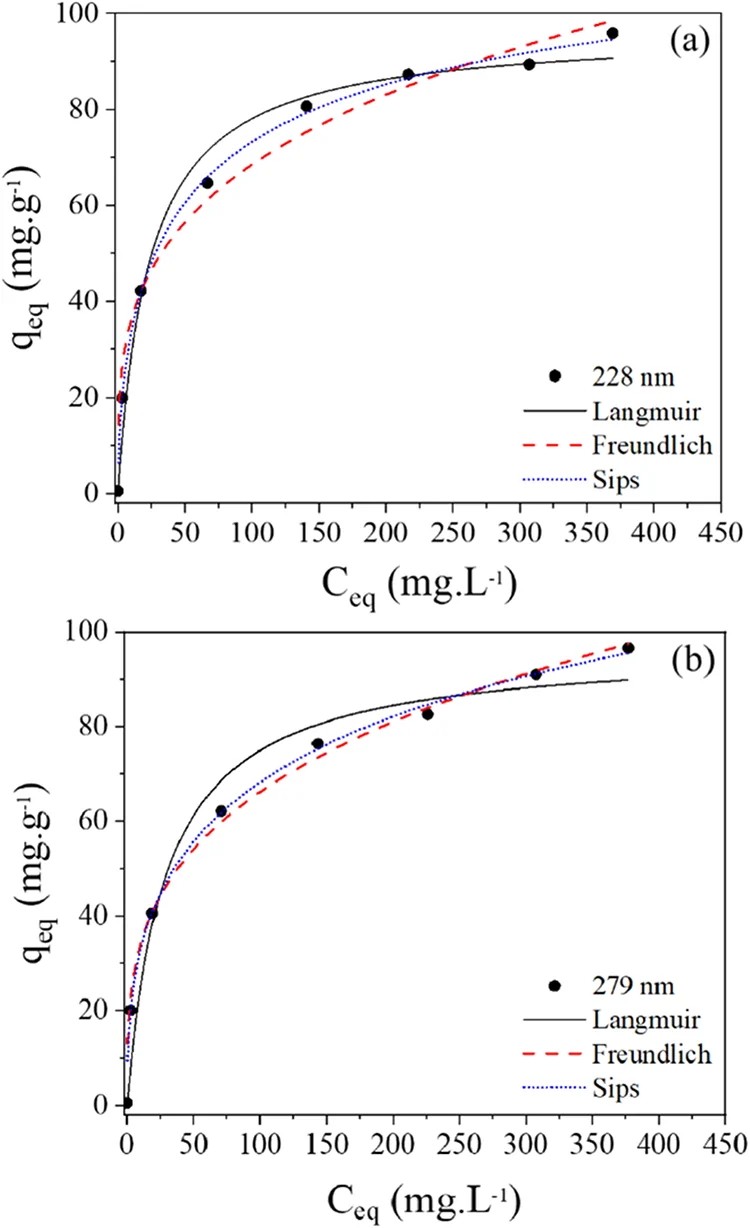
Fit of experimental data to adsorption models at (a) 228 nm and (b) 279 nm.

The adsorption isotherms obtained for mixture (Figure 5) presented behavior consistent with type L1, according to the classification proposed by Giles et al.^55^ This profile is characterized by a curve with concavity facing downward in relation to the concentration axis, indicating high affinity between adsorbed molecules and CA_H_3_PO_4__ surface, in addition to suggesting a predominance of contaminant–adsorbent interactions.^56^

It is also observed that as the initial concentration increased, the adsorption capacity followed the growth; this effect is attributed to the higher concentration gradient, which intensifies the driving force of the process until the occupation of the available active sites led to the establishment of equilibrium. The unknown parameter values obtained by fit of experimental data to Langmuir, Freundlich, and Sips models are presented in Table 5.

**Table 5:** Unknown Parameter Values Obtained by Fit of Experimental data to Langmuir, Freundlich, and Sips models

	Langmuir		Freundlich		Sips	
parameter	228 nm	279 nm	228 nm	279 nm	228 nm	279 nm
*q*_max_ (mg g^–1^)	96.38 ± 3.69	94.06 ± 3.04	-	-	127.49 ± 10.93	112.29 ± 6.67
*K*_L_ (L mg^*–*1^)	0.042 ± 0.008	0.046 ± 0.009	-	-	-	-
*K*_F_ (mg^–1^ L^1/*n*^)	-	-	18.99 ± 2.64	20.27 ± 3.21	-	-
*K*_S_ (L mg^*–*1^)	-	-	-	-	0.094 ± 0.009	0.096 ± 0.012
*n*	-	-	3.597 ± 0.026	3.846 ± 0.443	1.73 ± 0.15	1.54 ± 0.14
*R* ^2^	0.9834	0.9871	0.9872	0.9585	0.9985	0.9985
RSS	24.30	18.09	18.71	37.26	2.52	1.65

Based on Table 5, the adsorption models exhibited a good agreement with the experimental data (*R* ^2^ *>* 0.95); however, the Sips model presented the lowest RSS values, indicating superior fitting performance. The Sips model assumes that the adsorbent surface consists of homogeneous–heterogeneous adsorption sites.^57^ Under low solute concentrations, the adsorption behavior is well described by the Freundlich model, which accounts for surface heterogeneity and the presence of adsorption sites with different energy levels. Conversely, at higher concentrations, the adsorption process follows the Langmuir model, which assumes monolayer formation on the adsorbent surface.^58^ The values of *n* > 1 for Sips models indicate that the adsorption process is favorable for both pharmaceutical compounds.

Moreover, higher *n* values for Sips model were observed for the data obtained at λ = 228 nm, indicating a stronger interaction with CA_H_3_PO_4__. This behavior suggests a greater affinity of these compounds for the active sites on the adsorbent surface, resulting in an enhanced *q*_max_.

The adsorption potential of the activated biochar prepared from peanut shells (*q*_max_ ≥ 112 mg g^–1^) demonstrated an excellent adsorptive capacity when compared with several adsorbent materials commonly employed for the removal of PCM, PPN, and other pharmaceutical compounds. For instance, Tosin et al.^59^ prepared activated carbon from coconut shells for PCM removal and obtained a *q*_max_ of 39.68 mg g^–1^ within 49 min (*R* = 73.67%), considering an initial concentration range of 20–100 mg L^–1^.

Regarding more advanced adsorbents, such as green carbon nanotubes functionalized with oxygen-containing groups developed by Spaolonzi et al.^60^ for PPN removal, the authors reached *q*_max_ = 63 mg g^–1^ (*R* = 92.5%), over a concentration range of 13–220 mg L^–1^ after 360 min of contact time. In binary systems, such as the study conducted by Silva et al.,^61^ which investigated the adsorption of the pharmaceutical compounds ibuprofen and diclofenac using H_3_PO_4_-activated carbon produced from Brazil nut shells, *q*_max_ values of 59.6 and 42.2 mg g^–1^ were obtained for ibuprofen and diclofenac, respectively, within a concentration range of 3–50 mg L^–1^ after 180 min of contact time (*R* = 99%).

Subsequently, the effect of temperature on the adsorption process onto CA_H_3_PO_4__ was evaluated. Thus, the thermodynamic parameters Δ*G*, Δ*H*, and Δ*S* were determined at 298, 308, and 318 K. In Table 6 is shown the results of thermodynamic parameters.

**Table 6:** Thermodynamic Parameters Determined for the Adsorption of Mixture on CA_H_3_PO_4__

	Δ*G* (kJ mol^*–*1^)		Δ*H* (kJ mol^*–*1^)		Δ*S* (J mol^*–*1^ K^*–*1^)	
*T* (K)	228 nm	279 nm	228 nm	279 nm	228 nm	279 nm
298	–13.54 ± 0.29	–13.73 ± 0.66	-	-	-	-
313	–13.48 ± 0.30	–12.75 ± 0.66	–10.19 ± 2.62	–17.42 ± 9.68	11.22 ± 8.38	–13.19 ± 10.09
328	–13.86 ± 0.31	–13.39 ± 0.66	-	-	-	-
*R* ^2^	-	-	0.9397	0.7614	0.9397	0.7614
RSS	-	-	0.0048	0.0645	0.0048	0.0645

According to the results presented in Table 6, Δ*G* *<* 0 was observed at all temperatures and wavelengths investigated, confirming the spontaneous nature of the adsorption process. Furthermore, the relatively small variations of Δ*G* with increasing temperature suggest that the thermodynamic sensitivity of the system is limited, maintaining the spontaneous direction of the process under different conditions evaluated.

Regarding the Δ*H* values, the results indicate that the interaction between the pharmaceutical molecules and the CA_H_3_PO_4__ adsorbent occurs through an exothermic process. In agreement with these findings, Alawaideh et al.,^62^ while investigating the adsorption of PCM using H_3_PO_4_-activated carbon prepared from avocado waste, also reported a decrease in the equilibrium constant with increasing temperature, indicating the exothermic nature of the process. On the other hand, contrasting behaviors have been reported in the literature. Patel et al.,^63^ when evaluating the simultaneous adsorption of PCM and ciprofloxacin, observed that PCM exhibited an endothermic character, whereas ciprofloxacin showed an exothermic behavior. These findings demonstrate that thermodynamic behavior strongly depends on the specific interactions between contaminant molecules and the adsorbent surface, highlighting the importance of individual evaluation for each adsorption system.

The ΔS values indicated different behaviors depending on the wavelength considered. For λ = 228 nm, Δ*S* *>* 0 suggests an increase in disorder at the solid/liquid interface, attributed to the release of structured water molecules or counterions from the adsorbent surface during adsorption, leading to an overall increase in system entropy. In addition, in the binary system, competitive adsorption or displacement effects between the pharmaceuticals may contribute to this entropy gain, as rearrangement of adsorbed species can further enhance disorder, as reported by Obradovic et al.^64^ Conversely, for λ = 279 nm, Δ*S* *<* 0 was observed, indicating a decrease in randomness at the interface, possibly associated with a more ordered arrangement of pharmaceutical molecules on the CA_H_3_PO_4__ surface. This ordering may also reflect a specific affinity between the adsorbent and the adsorbate, as discussed by Al-Hazmi et al.^65^ The Δ*H* ∼20 kJ mol^–1^ indicate that the adsorption of the pharmaceutical compounds is predominantly physical in nature.^66^

In systems of this nature, the adsorption mechanism is likely associated with different types of intermolecular interactions. These include possible electrostatic interactions, arising from the attraction between the CA_H_3_PO_4__ surface and ionized pharmaceutical molecules; π–π interactions involving aromatic rings present in both the pharmaceutical compounds and the adsorbent structure; as well as the potential formation of hydrogen bonds with functional groups identified on the surface of the material.^53^

### Comparative Evaluation

In order to compare the efficiency of the prepared CA_H_3_PO_4__ adsorbent, the commercial adsorbent C_1012_ (Labsynth; characterization reported by Silva et al.^39^) was also evaluated (Table S7). The results are shown in Table 7.

**Table 7:** Comparison of the Performance of CA_H_3_PO_4__ and C_1012_ in the Removal of a Binary Pharmaceutical Mixture^a^

	228 nm		279 nm	
adsorbents	*q* (mg g^–1^)	*R* (%)	*q* (mg g^–1^)	*R* (%)
CA_H_3_PO_4__	5.20 ± 0.01	99.40 ± 0.10	5.29 ± 0.04	99.47 ± 0.70
C_1012_	5.16 ± 0.08	98.78 ± 1.62	5.32 ± 0.00	100.00 ± 0.00

a Conditions: *C*_0_ = 10 mg L^*–*1^, pH = 5.5, m/V = 2 g L^*–*1^, SS = 50 rpm, and t = 120 min.

From Table 7, it was observed that CA_H_3_PO_4__ and C_1012_ presented similar performance at the end of adsorptive process and exhibit comparable removal capacities. Furthermore, the CA_H_3_PO_4__ adsorbent demonstrated high viability and significant adsorptive potential in addition to its efficiency in the removal of pharmaceutical compounds from aqueous media. It is also worth emphasizing the relevance of using agroindustrial waste as a precursor material for adsorbent preparation, given the possibility of reintegrating such residues into the economic chain, thereby contributing to a more sustainable and circular approach to material utilization.

## Conclusions

The activated carbon obtained from peanut shells exhibited a high capacity for the adsorption of pharmaceuticals. Characterization analyses revealed that chemical activation promoted a significant increase in both surface area and pore volume compared to the precursor material as well as the formation of oxygenated functional groups on the adsorbent surface. Through the evaluation of contaminant removal, operational conditions were established that enabled the 100% removal of PCM and PPN in a binary mixture. The kinetic data demonstrated that adsorption equilibrium was achieved after 60 min, and the pseudo-second-order (PSO) model provided the best fit to the experimental data, indicating that the adsorption process is directly dependent on the availability of active sites. The adsorption of pharmaceutical compounds onto the surface of CA_H_3_PO_4__ occurs rapidly and is characterized by the presence of both homogeneous and heterogeneous sites with different energy levels, resulting in the formation of a monolayer on the adsorbent surface. Furthermore, the adsorbent exhibited a high availability of active sites, even at elevated contaminant concentrations. Therefore, the use of agroindustrial waste, such as peanut shells, represents a sustainable and promising alternative for the production of biosorbents. After undergoing appropriate physicochemical treatments, these materials develop structural characteristics that confer high adsorption capacity, demonstrating a strong potential for application in the treatment of wastewater contaminated with pharmaceutical compounds.

## Supplementary Material



## Notes

Complementary information regarding the preparation and evaluation of peanut shell-derived adsorbents for removing PCM and PPN. UV/Vis absorption spectra (Figure S1) and analytical calibration parameters (Table S1) for quantifying the binary mixture, detailed synthesis protocols for the five adsorbents, and the equations for the kinetic (Table S2), equilibrium (Table S3), and thermodynamic (Table S4) models, alongside linearized thermodynamic plots (Figure S2). Additionally, structural and textural characterizations are presented via Xray diffraction patterns (Figure S3) and pore size distribution charts (Figure S4), complemented by experimental performance data regarding the initial solution pH effect (Table S5), intraparticle kinetic parameters (Table S6), and adsorption isotherm fittings across different temperatures (Table S7) (PDF)
