# Supplementary material for: Valorization of Agroindustrial Waste: Use of Adsorbents from Peanut Shells for Removal of Paracetamol and Propranolol
Source: ACS Omega. 2026 Sep 15;11(38):56935–47. doi: 10.1021/acsomega.6c03834 (PMC13629033; doi:10.1021/acsomega.6c03834)
Supplement: Supplementary file 1 [file ao6c03834_si_001.pdf]

Valorization of agro-industrial waste: use of adsorbents from peanut shells for removal of paracetamol and propranolol

Raissa A. Freitas,[*,†] Ingrid L. S. Santana,[*,†] Marina N. S. Silva,[*,†] Giovanna A. J. C. Guedes,[*,†] Carolaine O. L. Silva,[*,†] João C. C. Rangel,[*,‡] Deivson C. S. Sales,[*,¶] and Marta M. M. B. Duarte[*,†]

†Departamento de Engenharia Química, Universidade Federal de Pernambuco, Recife, Pernambuco, Brazil.

‡Laboratório de Biocombustíveis (LAC/i-LITPEG), Universidade Federal de Pernambuco, Recife, Pernambuco, Brazil.

¶Escola Politécnica de Pernambuco, Universidade de Pernambuco, Recife, Pernambuco, Brazil.

E-mail: raissa.aguiarf@ufpe.br; ingrid.larissa@ufpe.br; marina.nuness@ufpe.br; giovanna.amaral@ufpe.br; carolaine.lima@ufpe.br; joao.rangel@ufpe.br; deivson.sales@upe.br; marta.duarte@ufpe.br

Valorization of agro-industrial waste: use of adsorbents from peanut shells for removal of paracetamol and propranolol

RAISSA AGUIAR DE FREITAS: raissa.aguiarf@ufpe.br

AFFILIATION: Departamento de Engenharia Química, Universidade Federal de Pernambuco, Recife, Pernambuco, Brazil.

ORCID: 0009-0009-5670-7359

INGRID LARISSA DA SILVA SANTANA: ingrid.larissa@ufpe.br

AFFILIATION: Departamento de Engenharia Química, Universidade Federal de Pernambuco, Recife, Pernambuco, Brazil.

ORCID: 0000-0001-8545-6637

MARINA NUNES DOS SANTOS SILVA: marina.nuness@ufpe.br

AFFILIATION: Departamento de Engenharia Química, Universidade Federal de Pernambuco, Recife, Pernambuco, Brazil.

ORCID: 0000-0002-8835-7308

GIOVANNA AMARAL JORGE CORREIRA GUEDES: giovanna.amaral@ufpe.br

AFFILIATION: Departamento de Engenharia Química, Universidade Federal de Pernambuco, Recife, Pernambuco, Brazil.

ORCID: 0009-0009-2928-9008

CAROLAINE DE OLIVEIRA LIMA SILVA: carolaine.lima@ufpe.br

AFFILIATION: Departamento de Engenharia Química, Universidade Federal de Pernambuco, Recife, Pernambuco, Brazil.

ORCID: 0009-0007-8952-532X

JOÃO CARLOS DA CUNHA RANGEL: joao.rangel@ufpe.br

Laboratório de Biocombustíveis (LAC/i-LITPEG), Universidade Federal de Pernambuco, Recife, Pernambuco, Brazil.

ORCID: 0009-0002-8029-8291

DEIVSON CESAR SILVA SALES*: deivson.sales@upe.br

Escola Politécnica de Pernambuco, Universidade de Pernambuco, Recife, Pernambuco, Brazil.

ORCID: 0000-0001-7879-0370

* Corresponding Author

MARTA MARIA MENEZES BEZERRA DUARTE: marta.duarte@ufpe.br

AFFILIATION: Departamento de Engenharia Química, Universidade Federal de Pernambuco, Recife, Pernambuco, Brazil.

ORCID: 0000-0002-5447-1380

Supporting Information S1

Figure S1. UV/Vis spectra of the (a) paracetamol (PCM), propranolol (PPN) and binary mixture (MB) and (b) their mixture different pH ranges for working solutions.

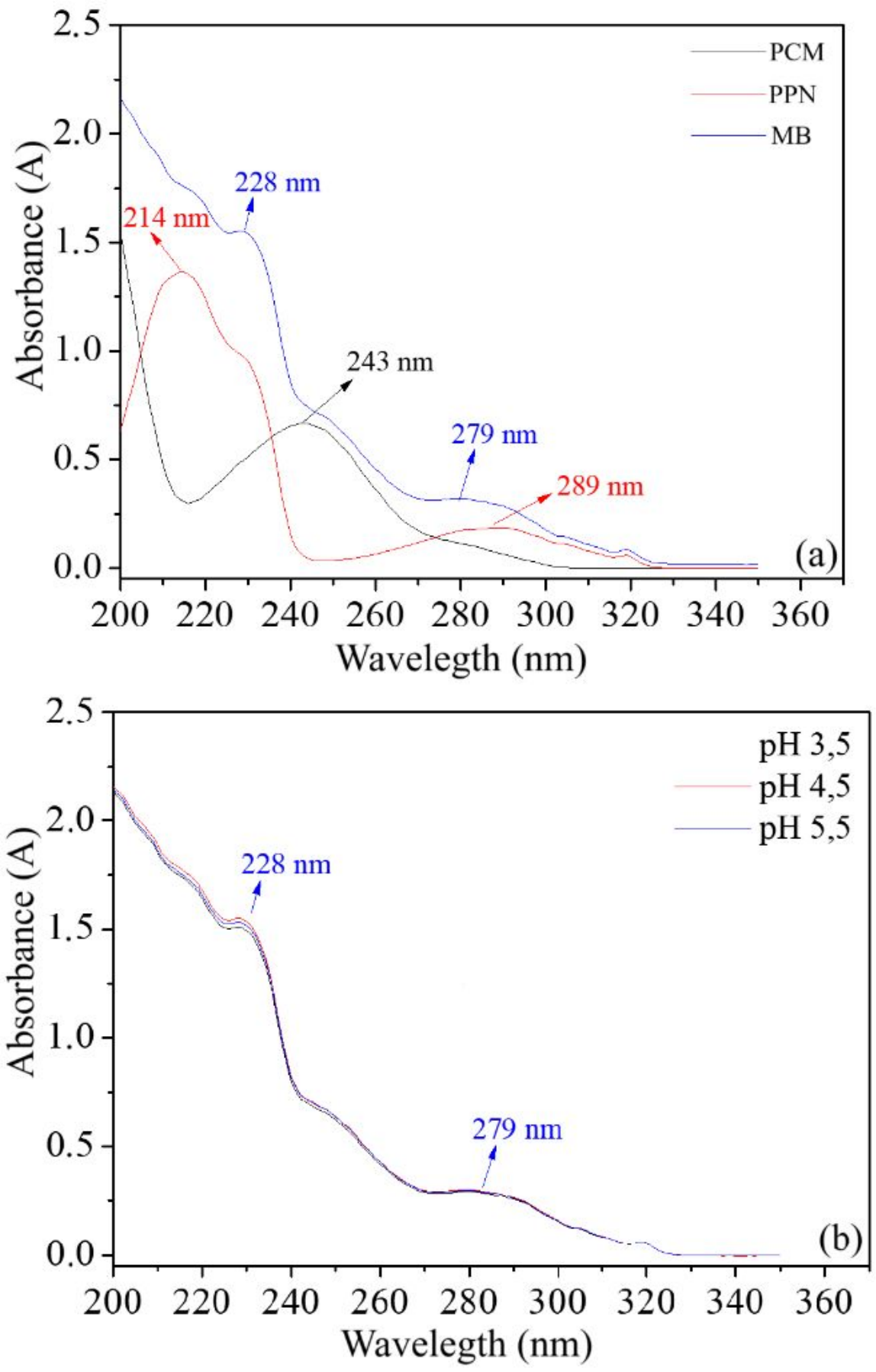

Table S1. Analytical curves for quantification of binary mixture of paracetamol and propranolol.

	λ (nm)	
Parameter	228	279
Equation	y = 0.1509991x	y = 0.02982661x
Limit of detection (LOD, mg□L^{-1})	0.06	0.08
Limit of quantification (LOQ, mg□L^{-1})	0.22	0.31
Coefficient of variation (COV, %)	2.03	3.10
Correlation coefficient (r)	0.99	0.99
Residual error	0.003	0.036

Supporting Information S2

Five adsorbents were prepared from peanut shell biomass:

In *natura material* (IN): The peanut shell was washed with running water and dried in an oven (brand: Quimis; model: Q31M-22) at 378 K for 24 h. After reaching room temperature (303 K), the material was crushed in a knife mill (brand: Cienlab; model: CE-430), washed with distilled water by immersion. After filtration, the material was dried again in the oven at 333 K for 24 h.

Basic washing (BW): The raw material was mixed, with the aid of a glass rod, with a solution of NaOH (0.1 mol L^{-1}) in a ratio of m/V = 1:20, and maintained for 2 h. After this period, the mixture was filtered (blue band filter paper) and the retained material was washed with distilled water by immersion. The washing process with distilled water was repeated until the solution reached a pH between 6 and 7. Then, the adsorbent was dried in the oven at 333 K for 24 h.

Activated carbon with H_3PO_4 (CA_{H3PO4}): The raw material was mixed, with the help of a glass rod, with H_3PO_4 in a ratio of m/V = 5:3. Carbonization occurred in an oven (brand: Quimis; model: Q318M24) with a heating ramp of 283 K min^{-1}, remaining at 373 K for 30 min, followed by 473 K for 1 h and 623 K for 1 h. After heat treatment, the material was washed by immersion with Na_2HCO_3 solution (1%) until the filtrate reached pH between 6 and 7, followed by a wash with distilled water. Finally, the activated carbon was dried in the oven at 333 K for 24 h.

CO_2 activated carbon (CA_{CO2}): For the pyrolysis process, 10 g of the raw material was placed in a quartz cylinder reactor (diameter: 2 cm diameter; length: 60 cm) and then in an electric furnace (brand: Thermo Scientific; model: Lindberg/Blue M) which was heated using a heating ramp from 283 K min^{-1} to 773 K, remaining so for 1 h, under 100 mL min^{-1} of N_2. After 1 h at 773 K, the flux of N_2 was replaced by CO_2 at the same flux rate for 1 h.

Functionalized (FC): The raw material was treated with HNO_3 in the ratio of m/V 5:3 for 3 h, followed by washing with NaOH (0.1 mol L^{-1}) to pH 7. It was then washed with distilled water by immersion. After filtering (blue band filter paper) and drying in an oven (brand: Quimis; model: Q318M24) at 333K for 24 h, the material was treated with 30 mL of H_2O_2 (35%) for 2 h, followed by filtration and drying at room temperature (303 K). This material was stored at 277 K.

After preparation, the materials were ground and sieved through a Tyler sieve, obtaining a grain size of less than 0.090 mm. All materials were then packed and stored in airtight containers, in order to ensure the integrity of their physicochemical properties and prevent possible external contamination.

Supporting Information S3

Table S2. Kinetic models.

Model	Equation	Parameter	Unit
PFO	$q_t = q_{eq}\,(1 - e^{-k_f t})$	q_t: adsorption capacity at t.	mg g^{-1}
		q_{eq} : adsorption capacity at equilibrium.	mg g^{-1}
		k_f : kinetic constant of PFO	min^{-1}
PSO	$q_t = \frac{k_s q_{eq}^2 t}{(1 + k_s q_{eq} t)}$	k_s : kinetic constant of PSO	g mg^{-1} min^{-1}
Weber and Morris	$q_t = K_d t^{0.5} + C$	K_d: intraparticle diffusion coefficient	mg g^{-1} $min^{-0.5}$
		C: constant related to diffusion strength, representing the thickness of the boundary layer.	mg g^{-1}
Boyd	$F = 1 - 6/\pi^2 \sum_1^0 1/n^2 \exp(-n^2 B_t)$	$F(q_t/q_{eq})$: adsorption fraction at t	-
	$F > 0.85 \Rightarrow B_t = -0.4977 - \ln(1 - F)$	B_t: mathematical function of F	-
	$F < 0.85 \Rightarrow B_t = \sqrt{\pi} - \left\{\sqrt{\left[\pi - \left(\frac{\pi - F}{3}\right)\right]}\right\}^2$	B_o: linear coefficient of the curve	-
		D: diffusivity coefficient	cm^{-2} min^{-1}
	$B = (\pi^2 D)/d^2$	B: Boyd constant (slope coefficient)	-
		d: average diameter of the particles	cm^{-1}

Supporting Information S4

Table S3. Equilibrium models.

Model	Equation	Parameter	Unit
Langmuir	$q_{eq} = \frac{q_{max} K_L C_e}{1 + K_L C_e}$	q_{eq}: adsorption capacity at equilibrium.	
		C_e: concentration at equilibrium.	mg L^{-1}
		q_{max} : maximum adsorption capacity	mg g^{-1}
		K_L: equilibrium constant of Langmuir model	L mg^{-1}
Freundlich	$q_{eq} = k_F C_e^{n_F}$	K_F : equilibrium constant of Freundlich model	mol^{-1} $L^{(1/n_F)}$
		n_F: heterogeneity factor of Freundlich model	-
Sips	$q_{eq} = \frac{q_{max}(K_S C_e)^{n_S}}{1 + (K_S C_e)^{n_S}}$	K_S : equilibrium constant of Sips model	L mg^{-1}
		n_S: heterogeneity factor of Sips model	-

Supporting Information S5

Table S4. Thermodynamics equations.

Equation	Parameter	Unit
$\Delta G = -RT \ln K_C$	$\Delta G°$: Gibbs free energy	J mol^{-1}
	R: constant of ideal gas	J mol^{-1} K^{-1}
	T: temperature	K
$K_c = 55.5 \cdot 10^3 K_L m$	K_C: adsorption constant at T	-
	m: mass of adsorbent	g
	K_L: adsorption constant of Langmuir model	L mg^{-1}
$\ln K_C = \left(\frac{\Delta H}{R}\right)\frac{1}{T} + \frac{\Delta S}{R}$	ΔH: enthalpy of adsorption	J mol^{-1}
	ΔS: entropy of adsorption	J mol^{-1} K^{-1}
$\ln K_L = \left(\frac{Q}{R}\right)\frac{1}{T} + \ln K_{L,0}$	$K_{L,0}$: pre-exponential constant of K_L	L mg^{-1}
	Q: adsorption heat	J mol^{-1}

Initially, the K_c values were estimated for each temperature, considering a mass of 0.1 g. Based on the K_c values obtained, ΔG values were determined. In sequence, $\ln K_C$ versus $1/T$ line was built (Figures S4a and S4b), from which the angular coefficient to determine ΔH, and the linear coefficient to determine ΔS were obtained. The Q values were determined in a similar way to the previous one.

Figure S2. $\ln K_C$ and $\ln K_L \times 1/T$: (a) $\ln K_C \times 1/T$ for 228 nm; (b) $\ln K_C \times 1/T$ for 279 nm; (c) $\ln K_L \times 1/T$ for 228 nm; (d) $\ln K_L \times 1/T$ for 279 nm.

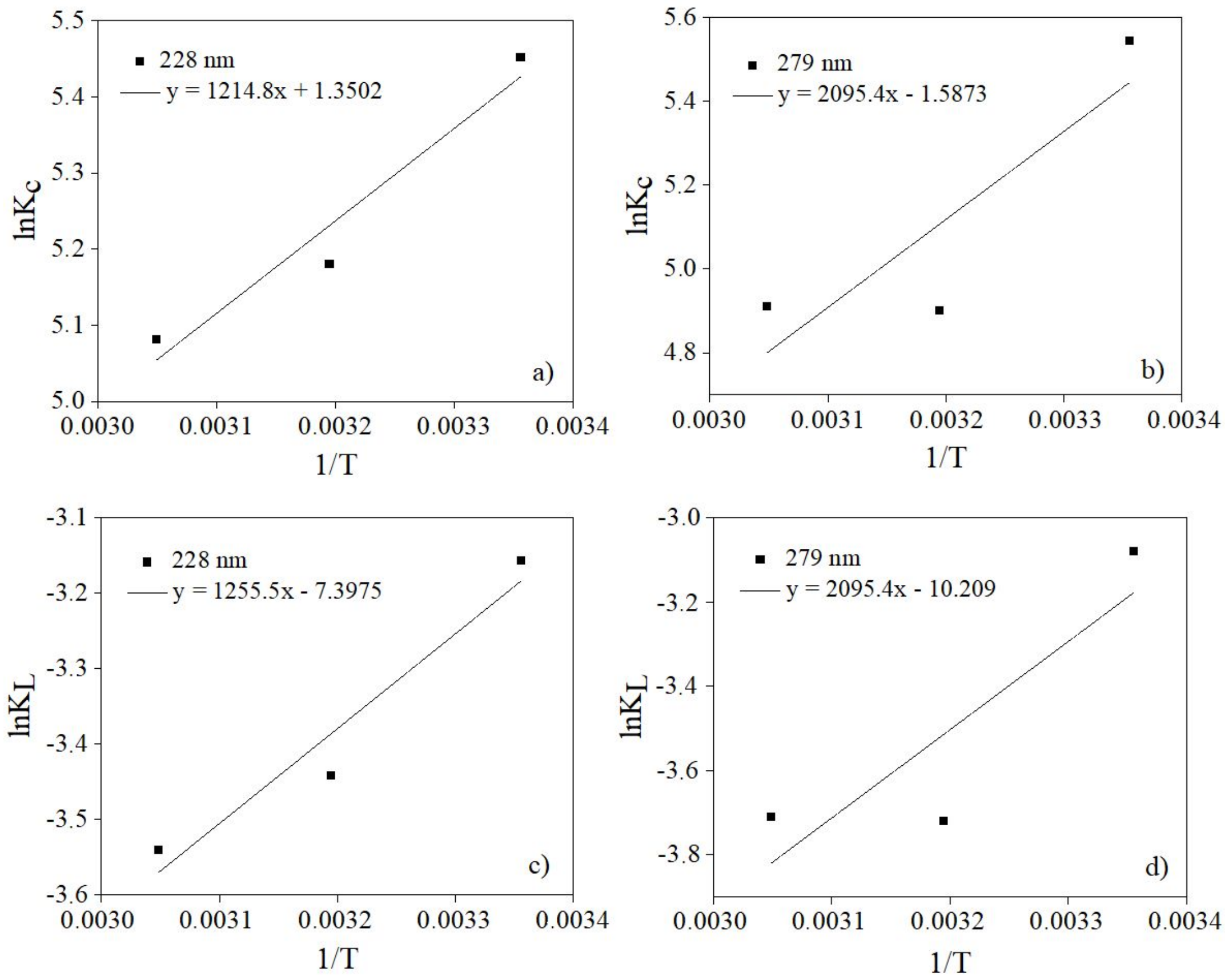

Supporting Information S6

Figure S3. X-ray diffractogram for IN and CA_{H3PO4}.

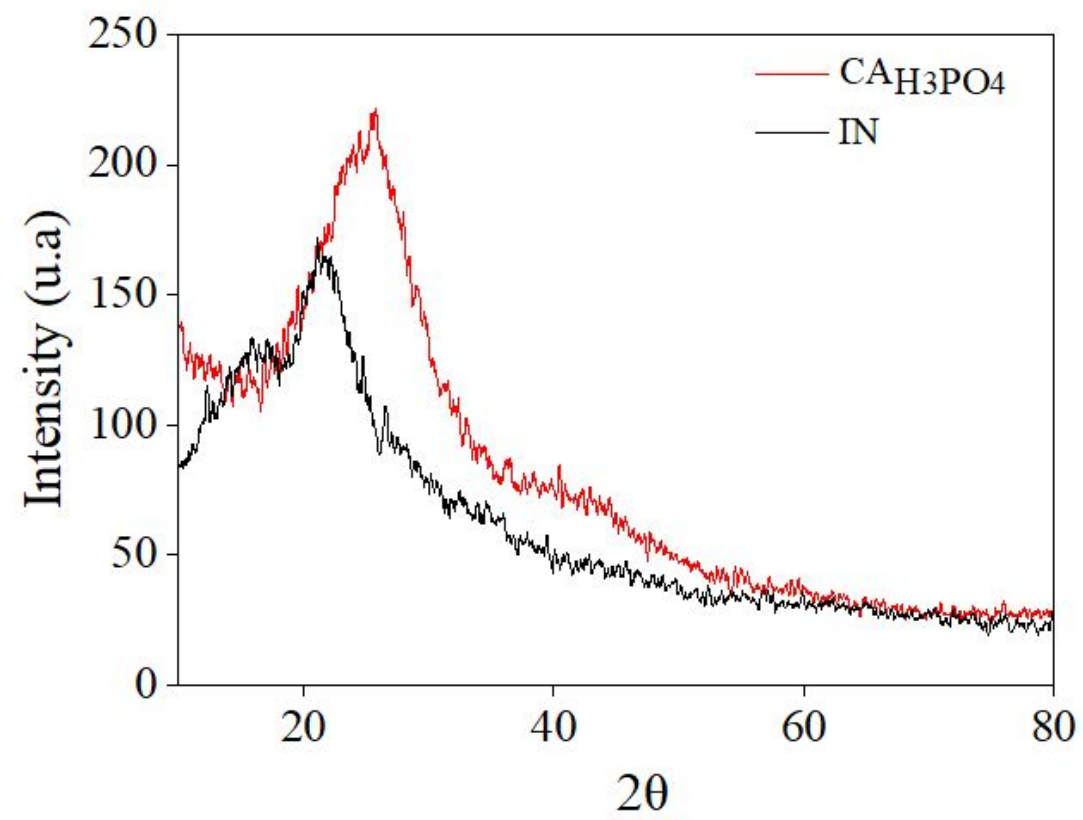

Figure S4. Pore distribution chart of the CA_{H3PO4}.

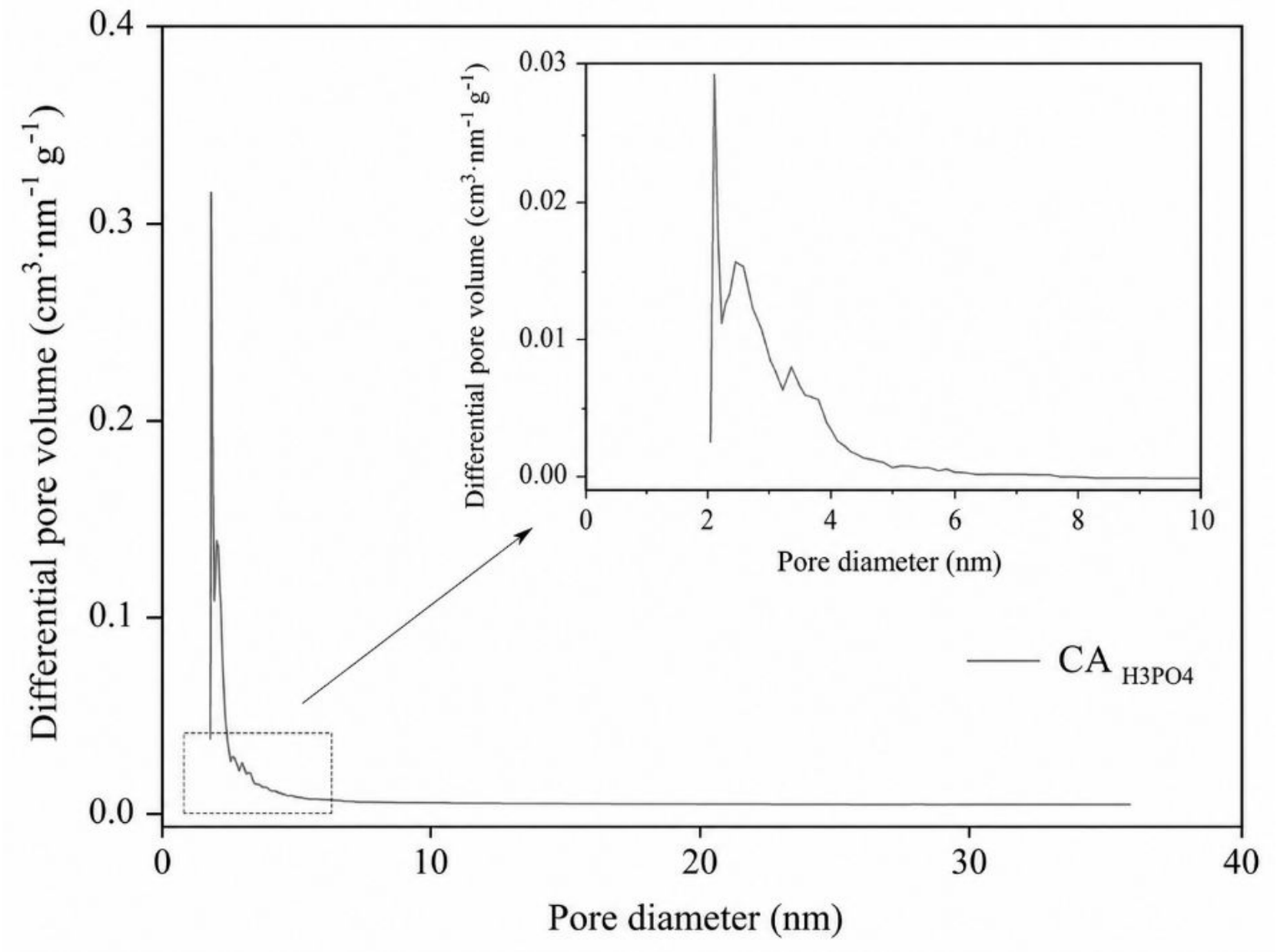

Supporting Information S7

Table S5. Effect of initial pH of solution. Conditions: C_0 = 10 mg L^{-1}; m/V = 2 g L^{-1}, t = 120 min, and T = 298 ± 2 K.

	228 nm		279 nm	
pH	q (mg g^{-1})	R %	q (mg g^{-1})	R %
3.5	5.07*	100*	5.33*	100*
4.5	5.07*	100*	5.27 ± 0.08	99 ± 1
5.5	5.05 ± 0.01	99.47 ± 0.09	5.33*	100*

* Presented no deviations.

Supporting Information S8

Table S6. Intraparticle kinetic models.

	228 nm		279 nm	
	Weber and Moris	Boyd	Weber and Moris	Boyd
Parameter				
K_d (mg g^{-1} $min^{-0.5}$)	0.05 ± 0.01	-	0.08 ± 0.01	-
C (mg g^{-1})	4.30 ± 0.02	-	4.10 ± 0.03	-
D (cm^{-2} min^{-1})	-	$2.19 \cdot 10^{-7}$	-	$2.04 \cdot 10^{-7}$
B_0	-	1.88 ± 0.07	-	1.57 ± 0.06
R^2	0.9828	0.9948	0.9838	0.9631

Supporting Information S9

Table S7. Adsorption isotherms.

	228 nm			279 nm		
Parameter	298 K	313 K	328 K	298 K	313 K	328 K
q_{max} (mg g^{-1})	96 ± 4	124 ± 4	128 ± 4	94 ± 3	125 ± 6	130 ± 4
K_L (L mg^{-1})	0.042 ± 0.009	0.032 ± 0.005	0.029 ± 0.004	0.046 ± 0.008	0.024 ± 0.005	0.024 ± 0.003
R^2	0.9834	0.9894	0.9924	0.9871	0.9846	0.9938
χ^2	24.30	24.17	18.37	18.09	33.69	14.69
